# A new ground beetle (Carabidae, Protorabinae) from the Lower Cretaceous of Inner Mongolia, China

**DOI:** 10.3897/zookeys.130.1300

**Published:** 2011-09-24

**Authors:** Bo Wang, Haichun Zhang

**Affiliations:** 1 State Key Laboratory of Palaeobiology and Stratigraphy, Nanjing Institute of Geology and Palaeontology, Chinese Academy of Sciences, 39 East Beijing Rd., Nanjing 210008, China

**Keywords:** Protorabinae, Coleoptera, Lower Cretaceous, Inner Mongolia, China

## Abstract

*Cretorabus rasnitsyni* **sp. n.**, belonging to the extinct subfamily Protorabinae of Carabidae, was described based on a well-preserved specimen from the Lower Cretaceous Yixian Formation of Yangshuwanzi, Inner Mongolia. The diagnostic characters for*Cretorabus* are revised, and the key to species of the genus was presented. The fossil record of Protorabinae was summarized. *Sinocarabus* Hong, 1982 and *Obesofemoria* Hong, 1982 cannot be attributed to Protorabinae.

## Introduction

Protorabinae is an extinct subfamily of Carabidae, and differs remarkably from all other carabid beetles in that the metepisterna are extending to the mesocoxal cavities (Ponomarenko 1977). The earliest Protorabinae, *Lithorabus incertus* Ponomarenko, 1977, was described from the Lower Jurassic of Issyk-Kul, Kirghizia (Ponomarenko 1977). Up to date, nearly 30 species within 8 genera are known from the Jurassic of Central Asia and the Lower Cretaceous of Mongolia, Russia, China and UK (Gromov et al. 1993; Ponomarenko 1977, 1980, 1986, 1989; Ponomarenko et al. 2005; Ren 1995). However, some Chinese Protorabinae require more detailed descriptions and revisions. The famous Early Cretaceous Jehol Biota yields abundant, well-preserved insects, including beetles. However, no record of Carabidae was reported from this lagerstätte until now. Herein, a new species of Protorabinae is described based on a well-preserved beetle from the Yixian Formation of Inner Mongolia, and the current fossil record of Protorabinae is summarized herein.

## Material and methods

The specimen (NIGP152464) was from the Lower Cretaceous Yixian Formation of Yangshuwanzi Village, Bisiyingzi Township, Ningcheng County, Chifeng City, Inner Mongolia. The fossils from Yangshuwanzi are commonly preserved in yellow silty mudstone. The horizon in this locality is equivalent to either the Jianshangou or Dawangzhangzi bed (about 124–122 Ma) of the Yixian Formation (Chang et al. 2009). The coleopteran assemblages from this locality were dominated by a variety of scarabs.

The specimen was examined dry and under alcohol, using a Nikon SMZ1000 stereomicroscope and drawings were made with the aid of a camera lucida. The photographs were prepared using a digital camera (DXM1200) connected to the above stereomicroscope, and the line drawings were readjusted on photographs using image-editing software (CorelDRAW X4 and Adobe Photoshop CS). In drawings, the broken lines denote the hidden and presumably missing body parts. Body length was measured along the midline from the anterior margin of frons to apex of elytra, and width was measured across the broadest part of elytra. The length of pronotum was measured along the midline; the width was measured across the broadest part of pronotum.

## Systematic Paleontology

**Family Carabidae Latreille, 1802**

**Subfamily Protorabinae Ponomarenko, 1977**

### Genus Cretorabus

Ponomarenko, 1977

http://species-id.net/wiki/Cretorabus

#### Type species.

*Cretorabus capitatus* Ponomarenko, 1977; by original designation.

#### Type horizon and locality.

Zaza Formation, Lower Cretaceous; Baissa, Buryatiya, Russia.

#### Diagnosis.

Body wide, small or medium-sized. Head large, strongly transverse. Pronotum transverse, widest in anterior or middle portion, constricted behind middle. Mesoventrite longer than mesocoxae. Metepisterna posteriorly tapering. Metacoxal plates tapering strongly in lateral half, extending as a narrow tongue up to lateral margins of metacoxae. Abdomen short, with rounded apex; last ventrite long, its anterior margin two-thirds narrower than base of abdomen. Legs short, femora slightly extending beyond body sides. Elytra smooth or with numerous rows of large punctures.

#### Remarks.

The genus is different from other genera in the metacoxal plates tapering strongly in lateral half, extending as a narrow tongue up to the lateral margins of metacoxae. Furthermore, it differs from *Cordorabus* Ponomarenko, 1977 in having the mesoventrite longer than mesocoxae, and abdomen short, with apex rounded; from *Ovrabites* Ponomarenko, 1977 in possessing the pronotum constricted behind middle, abdomen short, with apex rounded; from *Protorabus* Ponomarenko, 1977 by the last ventrite much narrower than base of abdomen, and elytra with grooves; from *Lithorabus* Ponomarenko, 1977 in having the metepisterna posteriorly tapering; from *Nebrorabus* Ponomarenko, 1989 in possessing the wider body and shorter legs.

#### Species included.

Six species: *Cretorabus capitatus* Ponomarenko, 1977 and *Cretorabus latus* Ponomarenko, 1977 from the Lower Cretaceous of Baissa; *Cretorabus orientalis* Ponomarenko, 1989 from the Lower Cretaceous of Khutel Khara of Mongolia; *Cretorabus ovalis* Ponomarenko, 1989 from the Lower Cretaceous of Bon-Tsagan of Mongolia; *Cretorabus sulcatus* Ponomarenko, Coram & Jarzembowski, 2005 from the Lower Cretaceous Purbeck Limestone Group of England; and *Cretorabus rasnitsyni* sp. n. from the Lower Cretaceous Yixian Formation of Inner Mongolia of China.

#### Key to species of *Cretorabus*

**Table:** 

1	Body small or medium-sized (length < 10 mm)	3
–	Body large ( > 13 mm)	2
2	Metacoxae 1.8 times as wide as long	*Cretorabus latus* Ponomarenko, 1977
–	Metacoxae 2.5 times as wide as long	*Cretorabus ovalis* Ponomarenko, 1989
3	Body small (length 3.7 mm); elytra with punctate furrows	*Cretorabus sulcatus* Ponomarenko, Coram & Jarzembowski, 2005
–	Body medium-sized; elytra smooth	4
4	Prosternal process narrow, 0.4 times as wide as procoxae, slightly longer than procoxae; metacoxae 1.5 times as wide as long	*Cretorabus capitatus* Ponomarenko, 1977
–	Prosternal process almost as wide as procoxae, much longer than procoxae; metacoxae 2.5 times as wide as long	5
5	Metaventrite twice as wide as long; metacoxal plates with emargination in lateral part of posterior margin	*Cretorabus orientalis* Ponomarenko, 1989
–	Metaventrite 3 times as wide as long; metacoxal plates with lateral part of posterior margin not emarginate	*Cretorabus rasnitsyni* sp. n.

### Cretorabus rasnitsyni

Wang & Zhang sp. n.

urn:lsid:zoobank.org:act:4416C789-0065-4CCC-9279-5F1419A1D481

http://species-id.net/wiki/Cretorabus_rasnitsyni

Figs 1 [Fig F2] –5

#### Holotype.

NIGP152464, male, a well-preserved beetle in ventral aspect. Yixian Formation, Lower Cretaceous; a fossil locality (41°25'N, 118°57'E) near Yangshuwanzi Village, Bisiyingzi Township, Ningcheng County, Chifeng City, Inner Mongolia, China. Deposited in the Nanjing Institute of Geology and Palaeontology (NIGP), Chinese Academy of Sciences.

#### Diagnosis.

Body medium-sized. Pronotum 1.8 times as wide as long. Prosternal process almost as wide as procoxae, much longer than procoxae. Metaventrite 3 times as wide as long. Metacoxae 1.8 times as wide as long. Metacoxal plates with lateral part of posterior margin not emarginate.

#### Description.

Head and pronotum strongly transverse. Length of head (including mandibles) slightly shorter than occiput width. Head capsule narrowing anteriorly from base. Eyes shorter than temples. Gular plate 1.5 times as long as wide, widened anteriorly. Antennae inserted at anterior margin of eyes; scape dilated; pedicellum slightly shorter than scape; first flagellomere slightly longer than scape. Mandibles large, slightly incurved, asymmetrical, with retinaculum small. Maxillary palps 4-segmented, conspicuously longer than mandibles; palpomere 4 with apical margin subtruncate.

**Figures 1–2.**
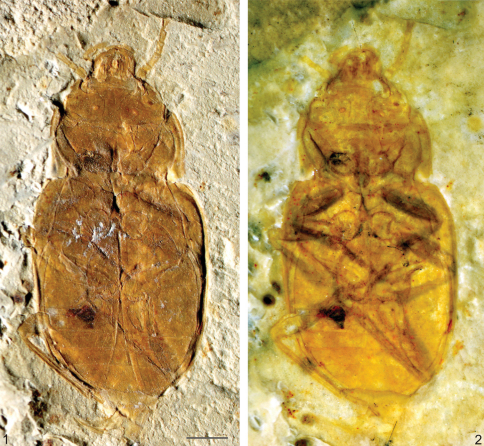
*Cretorabus rasnitsyni* sp. n., photomicrograph of holotype NIGP152464 **1** dry **2** under alcohol. Scale bar represents 1 mm.

Pronotum 1.8 times as wide as long, widest nearly in the middle; pronotal angles pointed, concealing the base of head dorsally. Propleura narrow. Prosternum before procoxae 1.4 times longer than procoxae. Prosternal process wide, 1.4 times as long as procoxae. Mesoventrite transverse, with subtriangular depression for reception of prosternal process. Mesepisternum almost rectangular, transverse. Mesepimeron short, extending to mesocoxal cavities, slightly widened laterally. Metaventrite short, 3 times as wide as long; its anterior margin half as long as the posterior. Metepisterna subtriangular, gradually widened anteriorly, its length 1.5 times width at anterior margin. Metacoxae oblique, slightly projecting over abdomen, 3 times as wide as long. Metacoxal plates slightly longer than coxae. Abdomen a little longer than meso- and metathorax combined, widened from base to apex of second visible sternite, then narrowing. Parameres rather long, with penis slightly projecting.

Profemur and protibia widened apically, almost equal in length. Tarsal segments widened apically; tarsomeres 1 almost as long as tarsomeres 2; tarsomeres 2 slightly longer than tarsomeres 3. Mesofemur and mesotibia almost equal in length. Mesotibia widened apically, 1.2 times as long as protibia. Metatrochanters one-third as long as metafemora. Metatibiae 1.1 times as long as metafemora, slightly widened apically; its outer side possibly with pits.

**Figures 3–4. F2:**
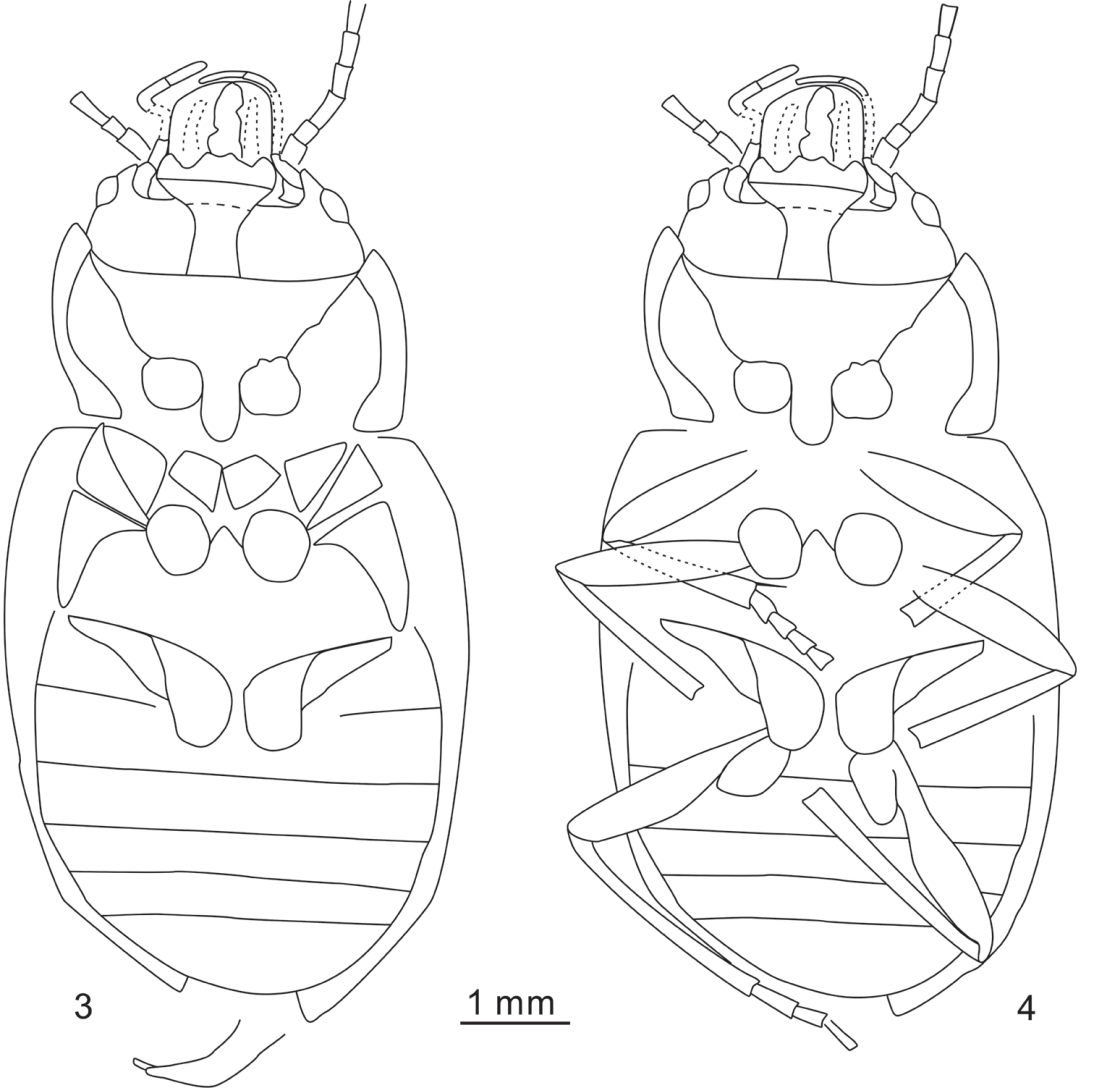
*Cretorabus rasnitsyni* sp. n., drawings of holotype NIGP152464 **3** without legs **4** with legs.

**Figure 5.**
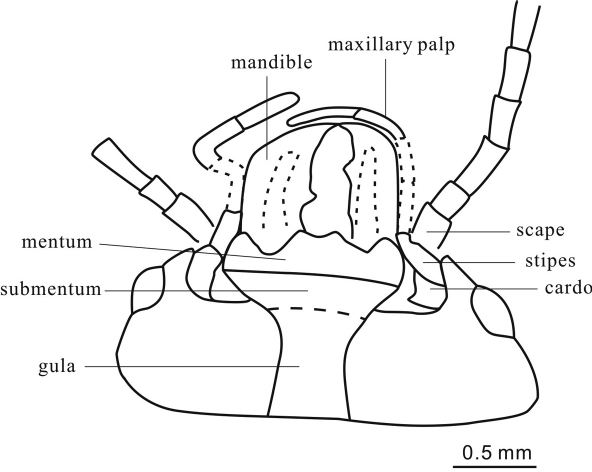
*Cretorabus rasnitsyni* sp. n., drawing of mouthparts of holotype NIGP152464.

#### Measurements.

Body length 7.8 mm, width 4.0 mm. Head length (including mandibles) 1.9 mm, occiput width 2.6 mm; mandible length 0.8 mm. Pronotum: length 1.8 mm, maximal width 3.2 mm. Abdomen: length 3.3 mm. Fore legs: femur length 1.5 mm; tibia length 1.5 mm. Middle legs: femur length 1.7 mm; tibia length 1.7 mm. Hind legs: femur length 2.0 mm; tibia length 2.3 mm.

#### Etymology.

Specific epithet is devoted to Alexander P. Rasnitsyn, an outstanding Russian palaeoentomologist.

#### Remarks.

This specimens can be undoubtedly attributed to *Cretorabus* by the following characters: the pronotum constricted behind middle; mesoventrite longer than mesocoxae; metacoxal plates tapering strongly in lateral half, extending as a narrow tongue up to the lateral margins of metacoxae; and abdomen short, with rounded apex. The species mostly resembles*Cretorabus orientalis* in having the prosternal process much longer than procoxae, and metacoxae 2.5 times as wide as long, but differs from the latter in possessing the metacoxal plates with the lateral part of the margin not emarginate. Furthermore, it is different from *Cretorabus capitatus* in having the comparatively wider prosternal process and metacoxae.

## Discussion

Ponomarenko and Kirejtshuk (2011) have revised and summarized the fossil record of Protorabinae, but the Chinese data require further revisions. Two monotypic genera, *Sinocarabus* Hong, 1982 and *Obesofemoria* Hong, 1982 were described from the Lower Cretaceous of Yumen of Gansu by Hong (1982), and also recorded in the list of fossil beetles by Ponomarenko and Kirejtshuk (2011). *Sinocarabus leptoceroides* Hong, 1982 was erected based on a specimen with the part and counterpart (Hong 1982). Judging from the original photograph (Hong 1982, plate 26, Figs 3, 4), this specimen seems to belong to Carabidae because of its body shape and strong mandibles. In the Hong’s original description, he stated that the “metacoxae round” and “metacoxae distant from each other”. Therefore, this specimen is not a carabid beetle. Moreover, Tan et al. (2004) also pointed out that the original drawing of this species showed some clear characters of Polyphaga. Thus, the systematic position of this specimen remains unclear. *Obesofemoria* Hong, 1982 was described based on a poorly-preserved specimen in dorsal aspect. This specimen does not show any diagnostic characters of Adephaga; thus more clear evidence is needed to resolve its taxonomic issue. Three specimens from the Lower Cretaceous Lushangfen Formation of Lushangfen of Beijing were transferred to Protorabinae by Ponomarenko and Kirejtshuk (2011): *Penecupes rapax* Ren, 1995, *Aethocarabus levigata* Ren, 1995, and *Nebrorabus tumoculus* (Ren, 1995). As a result, only four species, including the species described herein, within Protorabinae have been reported from the Mesozoic strata of China (Table 1). The diversity of Chinese Protorabinae based on the present data probably has been underestimated. More investigations on this topic should be done to understand better the true diversity of Protorabinae.

**Table 1.** Fossil record of Protorabinae.

Taxon	Occurrence
*Lithorabus incertus* Ponomarenko, 1977	Lower Jurassic of Issyk-Kul, Kirghizia
*Protorabus planus* Ponomarenko, 1977	Upper Jurassic of Karatau, Kazakhstan
*Protorabus magnus* Ponomarenko, 1977	Upper Jurassic of Karatau, Kazakhstan
*Protorabus nigrimonticola* Ponomarenko, 1977	Upper Jurassic of Karatau, Kazakhstan
*Protorabus kobdoensis* Ponomarenko, 1986	Lower Cretaceous of Myangad, Mongolia
*Protorabus crassus* Ponomarenko, 1989	Lower Cretaceous of Shiviya, Transbaikalia
*Protorabus tsaganensis* Ponomarenko, 1989	Lower Cretaceous of Bon-Tsagan, Mongolia
*Ovrabites ovalis* Ponomarenko, 1977	Upper Jurassic of Karatau, Kazakhstan
*Ovrabites jurassicus* Ponomarenko, 1977	Upper Jurassic of Karatau, Kazakhstan
*Ovrabites incertus* Ponomarenko, 1993	Lower Cretaceous of Khetana, Russian Far East
*Cordorabus notatus* Ponomarenko, 1977	Upper Jurassic of Karatau, Kazakhstan
*Cretorabus antennatus* Ponomarenko, 1977	Upper Jurassic of Karatau, Kazakhstan
*Cretorabus minimus* Ponomarenko, 1977	Upper Jurassic of Karatau, Kazakhstan
*Cretorabus vittatus* Ponomarenko, 1980	Lower Cretaceous of Manlay, Mongolia
*Cretorabus striatus* Ponomarenko, 1986	Lower Cretaceous of Gurban-Ereney, Mongolia
*Cretorabus capitatus* Ponomarenko, 1977	Lower Cretaceous of Baissa, Transbaikalia
*Cretorabus latus* Ponomarenko, 1977	Lower Cretaceous of Baissa, Transbaikalia
*Cretorabus orientalis* Ponomarenko, 1989	Lower Cretaceous of Khutel Khara, Mongolia
*Cretorabus ovalis* Ponomarenko, 1989	Lower Cretaceous of Bon-Tsagan, Mongolia
*Cretorabus sulcatus* Ponomarenko, Coram & Jarzembowski, 2005	Lower Cretaceous of Dorset, England
*Cretorabus rasnitsyni* sp. n.	Lower Cretaceous of Inner Mongolia, China
*Nebrorabus baculum* Ponomarenko, 1989	Lower Cretaceous of Chernyshevsk, Transbaikalia
*Nebrorabus capitatus* Ponomarenko, 1989	Lower Cretaceous of Baley, Transbaikalia
*Nebrorabus nebrioides* Ponomarenko, 1989	Lower Cretaceous of Bon-Tsagan, Mongolia
*Nebrorabus* ?*elongatus* (Ponomarenko, 1986)	Lower Cretaceous of Myangad, Mongolia
*Nebrorabus tumoculus* (Ren, 1995)	Lower Cretaceous of Lushangfen, China
*Aethocarabus levigata* Ren, 1995	Lower Cretaceous of Lushangfen, China
*Penecupes rapax* Ren, 1995	Lower Cretaceous of Lushangfen, China

## Supplementary Material

XML Treatment for Cretorabus

XML Treatment for Cretorabus rasnitsyni
